# Analysis of Argyrophilic Nucleolar Organizer Regions (AgNORs) in Acute Leukemia in Adults

**DOI:** 10.3390/diagnostics12040832

**Published:** 2022-03-28

**Authors:** Małgorzata Gajewska, Elżbieta Rutkowska, Iwona Kwiecień, Piotr Rzepecki, Kazimierz Sułek

**Affiliations:** 1 Department of Internal Medicine and Hematology, Military Institute of Medicine, Szaserów 128, 04-141 Warsaw, Poland; przepecki@wim.mil.pl (P.R.); sulekhem@poczta.onet.pl (K.S.); 2 Laboratory of Hematology and Flow Cytometry, Department of Internal Medicine and Hematology, Military Institute of Medicine, Szaserów 128, 04-141 Warsaw, Poland; erutkowska@wim.mil.pl (E.R.); ikwiecien@wim.mil.pl (I.K.)

**Keywords:** acute myeloid leukemia, acute lymphoblastic leukemia, argyrophilic nucleolar organizer regions, bone marrow, blasts

## Abstract

The evaluation of argyrophilic nucleolar organizer regions (AgNORs) uses a simple method used in research into neoplasm. Bone marrow aspirates from 70 patients with acute leukemia underwent morphological, immunophenotypic, and genetic assessment and were stained with silver nitrate. In leukemic cells, the mean AgNORs number, mean AgNORs area, and mean AgNOR-area-to-nucleus-area ratio were calculated in patients with acute myeloid leukemia (AML), patients with acute lymphoblastic leukemia (ALL), and selected risk groups. A higher value of all measured AgNOR parameters was observed in patients with AML compared to the ALL group. In AML patients, a higher mean AgNOR area was found in the ELN3 cytogenetic group compared to the ELN2 cytogenetic group. A higher value of the mean AgNOR count was observed in patients with white blood cells (WBCs) > 12 × 10^9^/L than in the group with WBCs ≤ 12 × 10^9^/L, as well as in patients with >20% blasts in peripheral blood (PB) than in patients with ≤20% blasts in PB. In the ALL group, a higher mean AgNOR-area-to-nucleus-area ratio was found in group with the presence of Philadelphia chromosome Ph(+) than without the Philadelphia chromosome Ph(−). AgNOR parameter analysis is a valuable method for differentiation of AML and ALL in adults.

## 1. Introduction

Nucleolar organizer regions (NORs) were first described by the German botanist Emil Heitz and the American geneticist Barbara McClintock as poorly stained regions around which nucleoli are renewed towards the end of the telophase [1].

These regions contain rDNA loops that constitute the matrix for rRNA synthesis. In humans, NORs are located on the short arms of five acrocentric chromosomes: 13–15, 21 and 22 [2]. With the use of the silver nitrate staining method, interphase NORs can be visualized under both optical and electron microscopes [2,3]. Silver-stained NORs are called argyrophilic nucleolar organizer regions (AgNORs), and the argyrophilic NOR proteins are called AgNOR proteins [1].

The first research results using the single-step silver staining technique proposed by Ploton for visualizing NORs were published as early as 1987, i.e., one year after the technique had been developed. The AgNOR staining method was used in research into neoplasms in organs and systems such as kidneys, prostate, bronchi, breasts, gastrointestinal tract, thyroid gland, uterus, and skin [4,5,6,7,8]. Most of the results, though not all, showed a correlation between the number of AgNORs in the cell and the survival of patients [9,10,11,12,13]. Ahmed H. G. et al. suggested the use of AgNOR protein analysis for predicting the expression of the estrogen receptor (ER), progesterone receptor (PR), and p53 protein, especially in countries with low socioeconomic status, where some of these markers can be unavailable in routine histopathology [14].

Nikicz and Norback used the single-step silver staining technique proposed by Ploton for visualizing NORs in normal bone marrow cells. They demonstrated that each type of bone marrow cell has specific AgNOR structures and that the shape and size of the AgNORs are correlated with the transcriptional and proliferative cell activity [15].

In case of non-Hodgkin lymphomas, initial research showed some correlation between the AgNOR proteins and the degree of malignancy, the percentage of cells with surface expression of Ki-67 protein, patients’ survival, remission occurrence, and remission duration [16,17,18,19,20,21,22,23]. The results of research conducted among patients with Hodgkin’s lymphoma suggested that determining the number of AgNORs may constitute a useful prognostic index, since the HL cells containing a larger number of AgNORs showed more sensitivity to the therapy administered [24]. Initial studies conducted in children with acute lymphoblastic leukemia (ALL) demonstrated that the group of patients with larger AgNOR surface areas was characterized by a higher number of deaths, more frequent relapses, and a shorter time until disease recurrence [9]. Studies conducted among patients with acute myeloid leukemia (AML) demonstrated that the mean number of AgNOR proteins in the myeloblastic cell was dependent on the type of leukemia, according to the French–American–British Classification (FAB). The patients in whom remission was obtained additionally demonstrated a larger number of AgNORs in their cells, compared to patients in whom remission was not achieved [10]. In their research based on the analysis of patient groups with different types of leukemias, Skonieczka et al. suggested that the numbers and surface areas of AgNORs can be used to differentiate between chronic lymphocytic leukemia (CLL) and chronic myeloid leukemia (CML), as well as between AML and ALL, and to differentiate between particular stages of CML [25]. The above analysis of selected AgNOR indexes in bone marrow cells of patients with different types of leukemia is one of the most recent publications.

Since available data are limited, in this research we analyzed selected parameters of AgNORs in bone marrow cells of AML and ALL patients, to search for characteristics allowing for the differentiation of these two leukemia types.

## 2. Materials and Methods

### 2.1. Group Characteristics

The study group consisted of 70 patients diagnosed, hospitalized, and treated at the Department of Internal Medicine and Hematology, Military Institute of Medicine, between 2017 and 2021. Patients who fulfilled the criteria of a blastic crisis diagnosis in chronic myeloid leukemia, patients with leukemia preceded by myeloproliferative neoplasm, and patients with a history of chemotherapy and/or radiotherapy due to solid tumors were excluded from the analysis.

Among the 45 patients with acute myeloid leukemia, there were 25 women and 20 men aged 20–85 (mean age 55.8). Based on the French–American–British criteria, the above group included 5 patients with M0 (11.1%), 20 patients with M2 (44.4%), 17 patients with M4 (37.8%), and 3 patients with M5 (6.7%). The patients were assigned to groups based on the cytogenetic–molecular classification developed by European Leukemia Net (ELN) in 2017: 27 patients were assigned to the cytogenetic–molecular risk group with intermediate prognosis (ELN2) and 18 patients to the cytogenetic–molecular risk group with poor prognosis (ELN3) [26].

Patients with acute lymphoblastic leukemia accounted for 25 cases: 11 women and 14 men aged 25–81 (mean age 55). Among the 21 patients with B-cell ALL, 10 had the confirmed presence of the Philadelphia chromosome.

Additionally, the following groups were distinguished on the basis of differences in sex, age, diagnosis, leukocyte level (WBC), hemoglobin level (HGB), PLT, blast percentage in peripheral blood, and blast percentage in bone marrow, in both subtypes of leukemia.

In order to classify by leukemia type and risk group, morphological, immunophenotypic, and genetic evaluations (bone marrow and/or peripheral blood) were conducted before any therapeutic intervention.

### 2.2. AgNOR Analysis

The bone marrow aspirate, dried at room temperature, was embedded in ethyl alcohol for 10 min. The preparation was then stained for 20 min at room temperature, without access to light, in a solution obtained by immediate, fast mixing of one volume of solution A (2% solution of gelatin dissolved in distilled water, to which formic acid was added until a 1% concentration final solution was obtained) with two volumes of solution B (50% solution of silver nitrate in distilled water). After staining, the preparations were placed for 10 min in a 5% sodium thiosulfate solution, and then rinsed in distilled water. The stained and dried slides were evaluated under an optical microscope with total magnification ×1000 (Olympus BX51 microscope, MDOB3 model, Tokyo, Japan). In each case, 200 cells corresponding to blast cells that were easy to identify (nuclei stained yellowish-brown and the AgNOR structures within the nuclei stained dark brown), were analyzed using a computerized image analysis system called cell* Soft Imaging System (Germany) and by the Microsoft Excel program. The following parameters were measured: the mean number of AgNORs in the nucleus, the mean surface area of AgNORs, and the ratio of AgNOR surface area to cell nucleus surface area. For each selected blast cell, the nucleus and each selected AgNOR structure were outlined. Then the data were collected, calculated using the author’s program, and sent to Excel.

### 2.3. Statistical Analysis

The following null hypothesis was considered. “There were no differences between the population means of selected characteristics in relation to the mean surface areas of AgNORs, the mean numbers of AgNORs, and the mean ratios of AgNOR surface area to nucleus surface area.” The alternative hypothesis was a negation of the null hypothesis.

The calculated significance levels for the tested characteristics (*p*-values) are summarized in the tables below. A value higher than the critical significance level (*p* < 0.05) did not justify the rejection of the null hypothesis.

The statistical analysis was conducted using Python 3.8 and Statistica 13.3 software (Statsoft, TIBCO Software Inc., Dell Inc., Palo Alto, CA, USA).

## 3. Results

**Figure 1:**
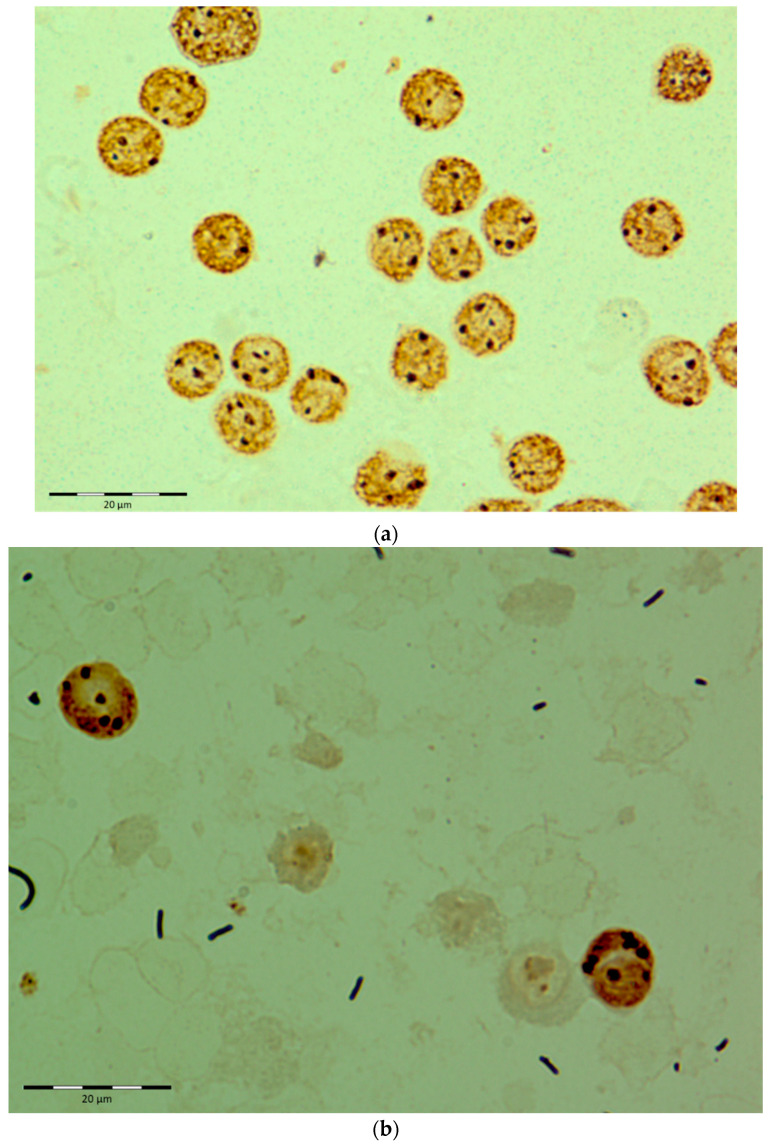

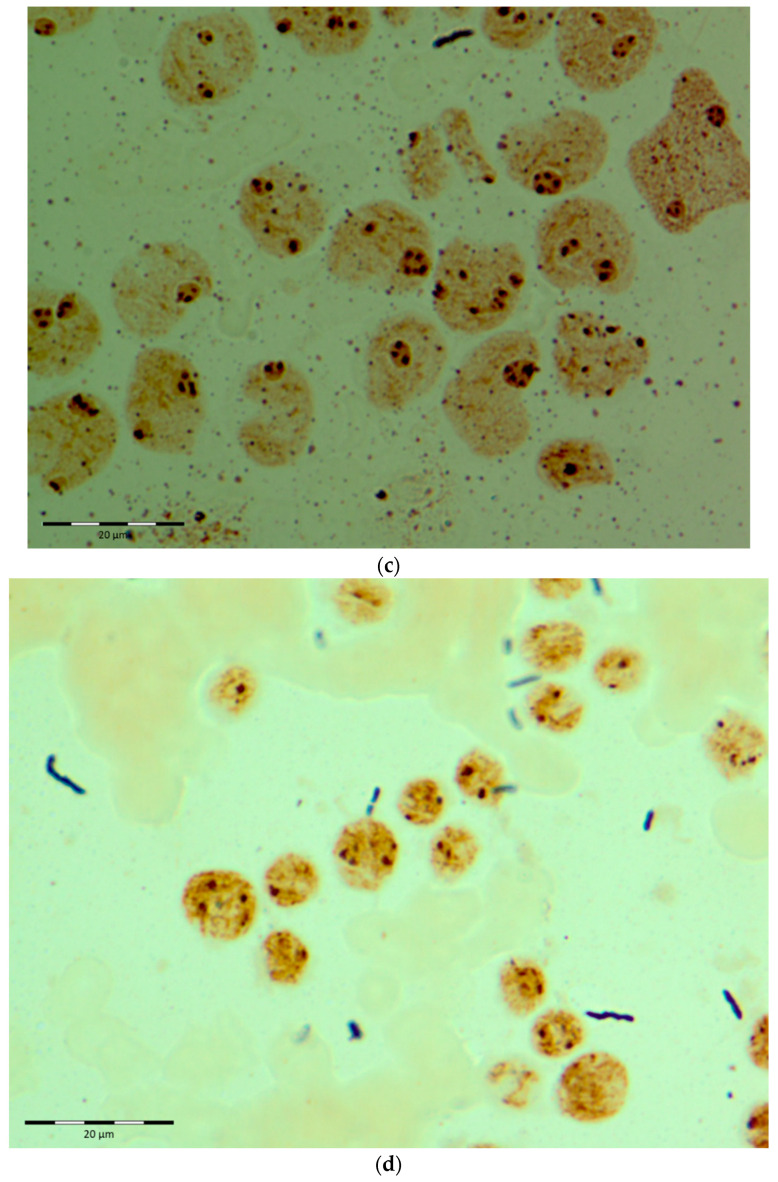
Exemplary results for AgNOR structure staining on bone marrow smears of selected patients: (**a**) M2 acute myeloid leukemia (ELN2); (**b**) M1 acute myeloid leukemia (ELN2); (**c**) M4 acute myeloid leukemia (ELN3); (**d**) acute lymphoblastic leukemia (Philadelphia+).

In leukemic cells, AgNOR parameters were calculated in patients with AML and ALL. Figure 1a–d shows exemplary results of AgNOR structure staining on bone marrow smears of selected patients.

### 3.1. Comparison of AML and ALL Groups

**Figure 2:**
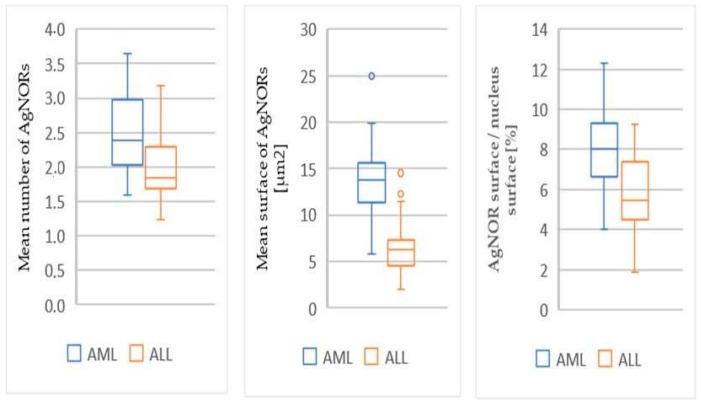
AgNOR indexes differences between AML and ALL: the mean number, mean surface area, and mean ratio of AgNOR surface area to nucleus surface area.

Significantly higher values of AgNOR parameters were found in patients with AML compared to the group with ALL, in the mean number of AgNORs (*p* < 0.001), mean surface area of AgNORs (*p* < 0.001), and mean ratio of the AgNOR surface area to the nucleus surface area (*p* < 0.001) (Table 1, Figure 2).

### 3.2. AgNOR Parameters in AML

We observed a higher value of mean AgNOR area in patients from the ELN3 cytogenetic group compared with patients from the ELN2 cytogenetic group (15.25 vs. 12.48; *p* = 0.02). A statistically significant difference in the mean AgNORs count was observed in patients with white blood cells (WBC) > 12 × 10^9^/L than in the group with WBC ≤ 12 × 10^9^/L (2.65 vs. 2.29; *p* = 0.04), as well as in patients with >20% blasts in peripheral blood (PB) than in patients with ≤20% blasts in PB (2.68 vs. 2.33; *p* = 0.01) (Table 2). No statistically significant differences were observed in the mean number of AgNORs, the mean surface area of AgNORs, or the mean ratio of AgNOR surface area to nucleus surface area depending on sex, age, FAB classification group, hemoglobin level, platelet count, leukocyte count, or blast percentage in peripheral blood (Table 2).

### 3.3. AgNOR Parameters in ALL

In the ALL group, a higher mean AgNOR-area-to-nucleus-area ratio was found in the group with the presence of Philadelphia chromosome Ph(+) than in the group without the presence of Philadelphia chromosome Ph(−) (6.15 vs. 4.01; *p* = 0.03). No statistically significant differences were observed in the mean number of AgNORs, the mean surface area of AgNORs, or the mean ratio of AgNOR surface area to nucleus surface area depending on sex, age, leukemia type group, WBC count, hemoglobin level, platelet count, or blast percentage in bone marrow and peripheral blood (Table 3).

## 4. Discussion

Nuclear organizing regions (NORs) are DNA strands involved in ribosome synthesis [27]. NORs are stained with silver nitrate under appropriate conditions, and the visualized structures are referred to as AgNORs, identified as black dots in the nuclei [28]. According to the literature, the most commonly used diagnostic index in bone marrow proliferative diseases is the number of AgNORs [25,29,30,31,32,33]. The AgNOR staining technique used for the assessment of nucleolar organizer regions is simple, quick, and cost-effective. It was used by Klobusicka et al. for determining the stage of the disease in different types of leukemia. The diagnostic value of the silver staining method and its potential significance in assessing cell proliferation in ALL, AML, and CML was assessed. It was found that, in remission, the mean number of AgNORs in the nucleus was lower than at diagnosis. An increase in the number of AgNORs was associated with relapse or blastic crisis in CML [29]. This parameter was also used by Nakamura et al. for comparison of AML and ALL [34]. Skonieczka et al. demonstrated that both the mean number of AgNORs and the mean surface of AgNORs were highest in acute leukemias (mostly in AML) and in patients at the blastic crisis stage of CML. It was demonstrated that the distribution of the mean sum of the AgNOR surface areas in the cell nucleus was similar to the distribution of the number of AgNOR granules. Therefore, the authors suggested considering not only the number of AgNOR granules but also the total surface area they occupy in the cell nucleus when analyzing the nucleolar organizer regions (NORs) in bone marrow proliferative diseases [25]. In our own research, the following parameters were measured in both types of leukemia: the mean number of AgNORs, the mean surface area of AgNORs, and the ratio of AgNOR surface area to nucleus surface area.

Our analysis showed a statistically higher mean number of AgNORs, mean surface area of AgNORs, and mean ratio of AgNOR surface area to nucleus surface area in the cell nucleus in the group of patients with AML compared to the group with ALL. These results coincide with the results obtained by Klobusicka et al., who also demonstrated a statistically higher mean number of AgNORs in AML [31]. Nakamura et al. did not demonstrate any difference between AML and ALL; however, a higher number of AgNORs was observed in AML [16]. In our research, we also analyzed the mean surface area of AgNORs and the ratio of the AgNOR surface area to the nucleus surface area. The distribution of the obtained data was similar to the distribution of AgNOR granules. These tests showed that all the indexes analyzed in this study can be used for the differentiation of AML and ALL.

The results obtained for the mean number of AgNORs coincide with the results obtained by Pich A. in a group of 40 patients with AML [35]. Like the above-mentioned study, our study did not demonstrate statistically significant differences in the mean number of AgNORs depending on sex, age, FAB classification group, cytogenetic group, hemoglobin level and platelets, or the number of blasts in peripheral blood. However, we observed a higher value of the mean AgNOR area in patients from the ELN2 cytogenetic group compared with patients from the ELN3 cytogenetic group. Furthermore, we showed a statistically significant higher value in the mean count of AgNORs in patients with white blood cells (WBC) > 12 × 10^9^/L than in the group with WBC ≤ 12 × 10^9^/L, and in patients with >20% blasts in peripheral blood (PB) than in patients with ≤20% blasts in PB.

In a group of patients with ALL B, a statistically significant difference between the mean ratio of the AgNOR surface area to the nucleus surface area was obtained in patients with the Philadelphia chromosome with a normal karyotype. By assessing a group of 36 patients with ALL, Pich A. et al. demonstrated a higher value of the mean number of AgNORs in patients with Ph+ [30]. As shown, the mAgNOR determination can be used as a quick, easy, and inexpensive prognostic factor for acute leukemia. However, a thorough analysis of AgNOR structures should be performed, including the ratio of AgNOR surface area to nucleus area.

The usefulness of this parameter was also confirmed by other researchers assessing the stage of cancer, as well as its prognostic significance [36]. Their findings suggest that AgNOR numbers increase with grade and remain low in normal breast tissue. A possible explanation for this may be related to the difference in mitotic activity between normal breast cells and malignant IDCs. Tyagi K. et al. also concluded that AgNORs may be a good marker of cell proliferative activity in aggressive cystic lesions with a malignant potential [37].

As our study and the above-mentioned studies show, AgNOR analysis of smears represents a simple, sensitive, and cost-effective method for differentiating leukemic subtypes with significant prognostic value and could be a readily available alternative method for the analysis of proliferative diseases. Due to the development of techniques used in diagnostics related to microscopy, a new implementation of AgNOR quantification should be considered.

## 5. Conclusions

The evaluation of genetic disturbances in the leukemic clone constitutes the basis for the classification of acute leukemias and provides information on the prognosis. Therefore, we believe that the analysis of AgNOR indexes in acute leukemias, with respect to the defined cytogenetic–molecular disorders, could broaden our perspective on these diseases and allow patients to be assigned to a defined risk group without the need to conduct difficult and specialist tests.

## Figures and Tables

**Table 1:** AgNOR indexes in AML and ALL: the mean number, mean surface area, and mean ratio of AgNOR surface area to nucleus surface area, expressed as percentages in AML and ALL.

AgNOR Cell Indexes	AML	ALL	*p*
	(*n* = 45)	(*n* = 25)	
Mean number of AgNORs	2.48 ± 0.59	1.99 ± 0.52	*p* < 0.001
Mean surface of AgNORs [µm^2^]	13.59 ± 3.79	6.58 ± 2.99	*p* < 0.001
AgNOR surface/nucleus surface [%]	8.15 ± 1.95	5.80 ± 1.82	*p* < 0.001

Abbreviation: AgNORs—agyrophilic nucleolar organizer regions.

**Table 2:** Mean values of AgNOR indexes in the AML patient group.

	No.	Mean AgNOR Count	Mean AgNOR Area	Mean AgNOR Area/Nucleus Area %
Whole series	45	2.48 ± 0.59	13.59 ± 3.86	8.15 ± 1.95
Sex		*p* = 0.24	*p* = 0.19	*p* = 0.36
Female	25	2.57 ± 0.57	12.91 ± 4.41	7.9 ± 2.22
Male	20	2.38 ± 0.59	14.44 ± 2.59	8.45 ± 1.50
Age		*p* = 0.13	*p* = 0.73	*p* = 0.62
<=65	17	2.52 ± 0.58	13.67 ± 3.65	8.04 ± 2.00
>65	28	2.39 ± 0.62	13.40 ± 4.41	8.40 ± 1.80
FAB classification		*p* = 0.76	*p* = 0.50	*p* = 0.32
M0	5	2.34 ± 0.79	12.91 ± 3.37	7.08 ± 1.13
M2	20	2.42 ± 0.54	12.72 ± 3.16	7.90 ± 2.09
M4	173	2.55 ± 0.61	14.52 ± 4.56	8.80 ± 1.93
M5		2.76 ± 0.63	16.69 ± 2.96	7.85 ± 2.16
Cytogenetic risk		*p* = 0.23	* *p* = 0.02	*p* = 0.7
ELN2	27	2.56 ± 0.61	12.48 ± 3.61	8.06 ± 2.17
ELN3	18	2.37 ± 0.56	15.25 ± 3.63	8.28 ± 1.69
White blood cells (×10^9^/L)		* *p* = 0.04	*p* = 0.78	*p* = 0.54
<=12	22	2.29 ± 0.49	13.72 ± 4.30	7.96 ± 1.90
>12	23	2.65 ± 0.66	13.39 ± 3.42	8.33 ± 2.09
Hemoglobin (g/dL)		*p* = 0.81	*p* = 0.94	*p* = 0.82
<=9	21	2.57 ± 0.58	13.65 ± 4.26	8.23 ± 1.95
>9	24	2.46 ± 0.60	13.54 ± 3.51	8.08 ± 2.04
Platelet count (×10^9^/L)		*p* = 0.40	*p* = 0.52	*p* = 0.65
<=50	25	2.54 ± 0.65	13.24 ± 4.07	8.27 ± 2.31
>50	20	2.41 ± 0.52	14.02 ± 3.56	8.00 ± 1.50
% of blasts in peripheral blood		* *p* = 0.01	*p* = 0.57	*p* = 0.18
<=20	25	2.33 ± 0.48	13.00 ± 4.37	7.53 ± 1.71
>20	20	2.68 ± 0.65	14.33 ± 2.73	8.92 ± 1.96
% of blasts in bone marrow		*p* = 0.60	*p* = 0.53	*p* = 0.35
<=50	26	2.52 ± 0.54	13.86 ± 4.17	8.35 ± 2.00
>50	19	2.44 ± 0.67	13.21 ± 3.40	2.38 ± 0.59

*—*p* < 0.05, Abbreviation: AgNOR—agyrophilic nucleolar organizer region; ELN—European LeukemiaNet.

**Table 3:** Mean values of AgNOR indexes in the ALL patient group.

	No.	Mean AgNOR Count	Mean AgNOR Area	Mean AgNOR Area/Nucleus Area %
Whole series	25	1.99 ± 0.52	6.58 ± 2.99	5.80 ± 1.82
Sex		*p* = 0.09	*p* = 0.89	*p* = 0.82
Female	11	2.20 ± 0.50	6.67 ± 3.04	5.71 ± 1.12
Male	14	1.83 ± 0.48	6.50 ± 2.95	5.88 ± 2.21
Age		*p* = 0.22	*p* = 0.36	*p* = 0.11
<=55	13	1.89 ± 0.59	6.01 ± 3.31	5.30 ± 2.03
>55	12	2.11 ± 0.41	7.19 ± 2.45	6.35 ± 2.35
Leukemia type		*p* = 0.38	*p* = 0.45	*p* = 0.18
ALL–T cells	5	1.87 ± 0.79	5.20 ± 3.27	6.07 ± 1.03
ALL–B cells	20	2.02 ± 0.45	6.84 ± 2.86	6.21 ± 1.74
ALL–B-cell		*p* = 0.62	*p* = 0.33	* *p* = 0.03
Ph(−)	10	2.23 ± 0.55	7.21 ± 2.83	4.01 ± 2.42
Ph(+)	11	1.83 ± 0.17	6.50 ± 2.84	6.15 ± 1.45
White blood cells (×10^9^/L)		*p* = 0.83	*p* = 0.13	*p* = 0.14
<=12	18	1.93 ± 0.37	7.10 ± 3.19	6.17 ± 1.46
>12	7	2.16 ± 0.77	5.24 ± 1.81	4.87 ± 2.26
Hemoglobin (g/dL)		*p* = 0.74	*p* = 0.16	*p* = 0.89
<=9	7	2.05 ± 0.44	7.96 ± 4.30	5.89 ± 1.69
>9	18	1.97 ± 0.55	6.04 ± 2.04	5.77 ± 1.86
Platelet count (×10^9^/L)		*p* = 0.32	*p* = 0.89	*p* = 0.65
<=50	10	2.13 ± 0.54	6.47 ± 3.24	5.73 ± 2.09
>50	15	1.90 ± 0.49	6.65 ± 2.81	5.85 ± 1.61
% of blasts in peripheral blood		*p* = 0.39	*p* = 0.24	*p* = 0.2
<=15	13	2.04 ± 0.52	7.36 ± 3.36	6.20 ± 1.56
>15	12	1.94 ± 0.52	5.73 ± 2.24	5.37 ± 1.97
% of blasts in bone marrow		*p* = 0.90	*p* = 0.17	*p* = 0.52
<=70	11	1.93 ± 0.41	7.67 ± 3.88	6.09 ± 1.44
>70	14	2.04 ± 0.59	5.72 ± 1.57	5.58 ± 2.04

*—*p* < 0.05, Abbreviation: AgNOR—agyrophilic nucleolar organizer region.

## Data Availability

Not applicable.
